# Estimulación temprana en el hogar de infantes que asisten a un centro infantil* **

**DOI:** 10.15649/cuidarte.2142

**Published:** 2022-08-22

**Authors:** Luz Angélica Orozco Restrepo, María Fernanda Cardona Cañas, Freddy Andrés Barrios Arroyave

**Affiliations:** 1 Fundación Universitaria Autónoma de las Américas, Docente investigador, Facultad de Medicina, Pereira, Colombia. Email: luz.orozcor@uam.edu.co. Fundación Universitaria Autónoma de las Américas Fundación Universitaria Autónoma de las Américas Facultad de Medicina Pereira Colombia luz.orozcor@uam.edu.co; 2 Fundación Universitaria Autónoma de las Américas, Docente investigador, Facultad de Medicina, Pereira, Colombia. Email: maria.cardonacan@ uam.edu.co Fundación Universitaria Autónoma de las Américas Fundación Universitaria Autónoma de las Américas Facultad de Medicina Pereira Colombia maria.cardonacan@ uam.edu.co; 3 Fundación Universitaria Autónoma de las Américas, Docente investigador, Facultad de Medicina, Pereira, Colombia. Email: freddy.barrios@uam.edu.co Fundación Universitaria Autónoma de las Américas Fundación Universitaria Autónoma de las Américas Facultad de Medicina Pereira Colombia freddy.barrios@uam.edu.co

**Keywords:** Desarrollo infantil, Crianza del Niño, Relaciones Familiares, Conducta Infantil, Child Development, Child Rearing, Family Relations, Child Behavior, Desenvolvimento infantil, Educação Infantil, Relações familiares, Comportamento Infantil

## Abstract

**Introducción:**

En el ambiente del hogar se propician estilos de crianza, aprendizajes, actividades, experiencias y estímulos que modulan la estimulación del niño(a).

**Objetivo:**

Identificar el grado de estimulación temprana y su relación con variables de tipología familiar y participación en el cuidado de niños(as) entre 1 y 4 años de un centro de desarrollo infantil en Pereira, Colombia, en 2019.

**Métodos:**

Estudio transversal. Se realizó un muestreo tipo censo que incluyó a todos los niños y cuidadores, que cumplían con los criterios de selección (niños sin antecedente de patologías neurológicas, consentimiento informado de cuidadores). Se midieron variables sociodemográficas y de tipología familiar. Se empleó el Inventario de estimulación temprana en el hogar HOME45 (Home Observation for Measurement of the Environment). Se realizó análisis univariado y bivariado. Para la asociación entre predictores y el puntaje global de estimulación temprana (desenlace: alta/media/baja) se efectuó una regresión logística ordinal.

**Resultados:**

Participaron 76 diadas madre-hijo. La mediana de edad de los niños fue 36 meses (RIQ=11, 12-48). El cuidado diario fue brindado en un 67% por la madre. Se evidenció una alta estimulación en el 50% de las diadas. Los predictores que redujeron la probabilidad de estimulación alta fueron (p<0,05): hábito de lectura (No, RP=0,29 (0,09-0,87)), participación en fiestas infantiles (No, RP=0,24 (0,07-0,79)), edad del cuidador (mayor a 36 años, RP=0,95 (0,92-1,00)), estrategia de corrección (castigo verbal o físico, RP=0,16 (0,03-0,98)).

**Conclusión:**

Corregir al niño mediante diálogo, incentivar la lectura y participar de fiestas infantiles, además de tener un cuidador menor de 35 años, fueron variables que incrementaron la probabilidad de presentar una alta estimulación.

## Abstract (en)

**Introduction:**

Home environment fosters parenting styles, learning, activities, experiences, and stimuli modulating children’s stimulation.

**Objective:**

To identify the degree of home-based early stimulation and its relationship with family variables and participation in the care of children aged 1 to 4 years attending a daycare center in Pereira, Colombia in 2019.

**Materials and Methods:**

A cross-sectional study was conducted by means of a census sampling including all children and caregivers who met the selection criteria (children with no history of neurological disorders and caregiver informed consent.) Sociodemographic and family variables were also measured. The Early Childhood HOME45 (Home Observation for Measurement of the Environment) inventory was administered. Univariate and bivariate analyses were carried out. Ordinal logistic regression was performed for associating predictors and total early stimulation score (high/medium/low).

**Results:**

The average age of children was 36 months (RIQ=11, 12-48). 67% of mothers gave children daily care. High stimulation was observed for 50% of the dyads. Predictors that reduced the probability of high stimulation (p<0.05) were reading habit (No, PR=0.29 (0.09-0.87)), participation in children’s parties (No, PR=0.24 (0.07-0.79)), caregiver age (older than 36 years old, PR=0.95 (0.92-1.00)), child discipline strategy applied (verbal or physical abuse) (PR=0.16 (0.03-0.98)).

**Conclusions:**

Educating children through dialogue, encouraging reading and participating in children’s parties, as well as having a caregiver under 35 years of age, were variables that increased the probability of high stimulation in children.

## Resumo

**Introdução:**

O ambiente familiar fomenta estilos de criação, aprendizagem, atividades, experiências e estímulos que modulam a estimulação das crianças.

**Objetivo:**

Identificar o grau de estimulação precoce e sua relação com variáveis de tipologia familiar e participação no cuidado de crianças entre 1 e 4 anos de idade em um centro de desenvolvimento infantil em Pereira, Colômbia, em 2019.

**Métodos:**

Estudo de corte transversal. Foi realizada uma amostragem tipo censo que incluiu todas as crianças e cuidadores que preenchiam os critérios de seleção (crianças sem histórico de patologias neurológicas, consentimento informado dos cuidadores). As variáveis sociodemográficas e de tipologia familiar foram medidas. O inventário de estimulação precoce HOME45 (Home Observation for Measurement of the Environment) foi utilizado. Foram realizadas análises univariadas e bivariadas. Para a associação entre os preditores e a pontuação global de estimulação precoce (resultado: alto/médio/baixo), foi realizada uma regressão logística ordinal.

**Resultados:**

Setenta e seis díades mãe-filho participaram. A idade média das crianças era de 36 meses (RIQ=11, 12-48). O cuidado diário era de 67% fornecido pela mãe. A alta estimulação era evidente em 50% das díades. Os preditores que reduziram a probabilidade de alta estimulação foram (p<0,05): hábito de leitura (Não, PR=0,29 (0,090,87)), participação em festas infantis (Não, PR=0,24 (0,07-0,79)), idade do cuidador (mais de 36 anos, PR=0,95 (0,92-1,00)), estratégia de correção (castigo verbal ou físico, PR=0,16 (0,03-0,98)).

**Conclusão:** Corrigir a criança através do diálogo, incentivar a leitura e a participação em festas infantis, assim como ter um cuidador com menos de 35 anos de idade, foram variáveis que aumentaram a probabilidade de apresentar um alto nível de estimulação.

## Introducción

La primera infancia ha sido considerada la fase de desarrollo más importante de todo el ciclo vital, en ella se llevan a cabo importantes procesos de maduración y aprendizaje, a nivel cerebral hay desarrollo acelerado de sinapsis, proceso que se genera durante los primeros tres años de vida en respuesta a las experiencias percibidas del medio^1^^,^^2^^,^^3^.

Este desarrollo está determinado por influencias genéticas, biológicas y ambientales, destacándose como necesarias las interacciones tempranas, los estilos de crianza y la organización de un entorno físico y humano inmediato, siendo el entorno familiar donde se concentran y propician importantes aprendizajes que generan oportunidades para que ocurran actividades, experiencias, emociones y estímulos que modulan el desarrollo del niño y de la niña^4^^,^^5^.

A nivel mundial las cifras reportan un preocupante 43% de niños y niñas menores de 5 años (249 millones) que están en riesgo de un pobre desarrollo infantil debido a la situación de pobreza, maltrato, baja escolaridad de la madre y desnutrición crónica en que viven^6^.

En la mayoría de los países, menos de la mitad de los niños asisten a programas de enseñanza para la primera infancia y quedan privados de oportunidades de ingresar en la escuela 59 millones de niños en edad de acceder a la educación primaria. La deficiencia de oportunidades de aprendizaje es preocupante ya que el entorno escolar garantiza oportunidades de aprendizaje y adquisición de conocimientos básicos. Las experiencias en el hogar son determinantes para el éxito en el aprendizaje, ligadas a la presencia de un entorno familiar estimulante^7^^,^^8^. Estudios apoyan que los ambientes enriquecidos culturalmente pueden asociarse a mejores rendimientos en el campo cognitivo y académico^9^^,^^10^, estos resultados refuerzan la recomendación sobre el apoyo de la educación infantil en contextos familiares, educativos y la evaluación constante del desarrollo^11^.

El presente estudio busca determinar el grado de estimulación temprana en el hogar y su relación con variables de tipología familiar y participación en el cuidado de los niños y niñas entre 1 y 4 años que asisten a un centro de desarrollo infantil público del Instituto Colombiano de Bienestar Familiar (ICBF) en Pereira, durante el 2019-2020

## Materiales y Métodos

Estudio transversal en el que se incluyó a un grupo de niños y sus cuidadores. Los datos se recolectaron entre abril del año 2019 y junio del año 2020. La población objetivo era de 120 niños y sus cuidadores, de los cuales 76 cumplieron con los criterios de selección, utilizando para la caracterización sociodemográfica y la descripción de las variables inherentes a la tipología familiar, un cuestionario de construcción propia, el cual se avaló mediante prueba piloto y por consulta a expertos.

Para evaluar el grado de estimulación en el hogar se utilizó el inventario HOME45^12^. La encuesta se aplicó al cuidador principal, y se observaron las conductas de la diada infante/cuidador. Se incluyeron niños y niñas entre 1 y 4 años de edad, sin patologías neurológicas, con aceptación y firma del consentimiento informado por parte de sus tutores legales. Se realizó un censo incluyendo a toda la población objetivo, los infantes y cuidadores que asisten al Centro de Desarrollo y cumplieran con los criterios del estudio.

Aunque no está validado en Colombia, el instrumento HOME45 está respaldado por más de 600 trabajos publicados desde 1967 a la actualidad, la mayoría de ellos vinculándolo con el desarrollo, comportamiento o aprendizaje del niño y características socioeconómicas o psicosociales de la familia y los padres. Además, ha sido utilizado en diferentes estudios a nivel de Latinoamérica, en países como Chile^13^, Argentina^14^ y México^15^.

La versión original del inventario HOME para niños de cero a tres años publicada por sus autores Caldwell y Bradley contiene seis dimensiones y cuarenta y cinco reactivos. El instrumento evalúa cinco dimensiones: responsividad (conductas de la madre, 11 ítems), aceptación (de las conductas del niño, 8 reactivos), organización (del ambiente físico, 6 preguntas), materiales de juego (apropiados, 9 ítems), involucramiento (madre se involucra con el niño, 6 reactivos) y variedad (oportunidades de estimulación, 5 preguntas). Los resultados indican el grado de estimulación global en el hogar según la puntuación total (45 puntos posibles)^12^.

El alfa de Cronbach de la versión para infantes utilizada en este estudio, que está conformada por 45 reactivos, es de 0,773 en su puntuación total; y para cada una de las dimensiones: Responsividad (alfa: 0,709), Aceptación (alfa 0,621), Organización (alfa 0,421), Materiales de juego (alfa 0,648), involucramiento (alfa 0,661), Variedad (alfa 0,667)^16^.

Los ítems se puntean con 0 (-) y 1 (+) mostrando ausencia o presencia de lo estipulado en el ítem^17^. Los puntajes se categorizan según el puntaje total y por subescalas en nivel de estimulación alto, medio y bajo, los cuales tienen límites de acuerdo al rango de edad en que se encuentra el infante (medido en meses), estas puntuaciones se muestran en la Tabla 1.

**Tabla 1:** Puntos de corte para interpretación de resultados de aplicación del Inventario HOME-45 según puntuación total y por subescalas

Límites	Nivel estimulación	HOME Total	Responsividad	Aceptación	Organización	Materiales	Involucramiento	Variedad
Límite 1 (0-5 m)	Baja	0-22	0-6	0-5	0-3	0-1	0-1	0-1
	Media	23-28	7-9	6	4	2-3	2-4	2-3
	Alta	29-45	10-11	7-8	5-6	4-9	5-6	4-5
Límite 2 (6-11 m)	Baja	0-27	0-7	0-5	0-3	0-4	0-2	0-1
	Media	28-34	8-10	6	4-5	5-7	3-5	2-3
	Alta	35-45	11	7-8	6	8-9	6	4-5
Límite 3 (12-23 m)	Baja	0-27	0-7	0-5	0-2	0-4	0-2	0-1
	Media	28-34	8-10	6	3-4	5-7	3-5	2-3
	Alta	35-45	11	7-8	5-6	8-9	6	4-5
Límite 4 (24-40 m)	Baja	0-28	0-7	0-5	0-3	0-4	0-2	0-1
	Media	29-35	8-10	6	4	5-7	3-5	2-3
	Alta	36-45	11	7-8	5-6	8-9	6	4-5

Fuente: Datos obtenidos del Manual de Vigilancia integral del niño, México 2009

Se evaluaron adicionalmente variables sociodemográficas del niño y cuidador, tales como: edad, sexo, escolaridad del cuidador, y variables familiares como la tipología de cada hogar, personas al cuidado del niño/niña y actividades en casa. Las variables de estimulación en el hogar consideraron aspectos del grado de estimulación según cada una de las subcategorías, como ambiente en que juega el infante, juguetes utilizados, respuestas del cuidador, patrones de aceptación, variedad de estímulos, entre otros.

El presente trabajo no incluyó en su metodología una intervención educativa, pero a cada una de las diadas se le hizo entrega de material literario y explicación de trabajo con el niño en casa por medio de la implementación de hábitos de lectura, teniendo en cuenta que el instrumento considera la misma como un aporte importante al estímulo en el hogar. Teniendo en cuenta que el instrumento Home es complejo, se realizó estandarización de los evaluadores por medio de una prueba piloto previa a la recolección de información.

Se realizó un análisis exploratorio y descriptivo de los datos. Los resultados se expresaron por medio de tablas de frecuencias simples y porcentuales para las variables cualitativas y estadísticas de resumen (tendencia central y dispersión) para variables cuantitativas, según su ajuste e distribución (evaluada a través del test Shapiro Wilks). Para analizar la asociación entre variables independientes o predictores (“*Xi*”) y el puntaje HOME45 global, que fue identificado como variable dependiente o desenlace (“*Y*”), bajo la clasificación categórica ordinal: alta (*Y*_*1*_ *)*, media (*Y*_*2*_ *)*, baja (*Y*_*3*_ *)*, se llevaron a cabo regresiones logísticas ordinales simples, obteniendo razones de prevalencia (*RP*), odds ratio (*OR*) y valores *p* como medidas de asociación. Posteriormente, y de acuerdo con el cumplimiento de supuestos, las *Xi* que resultaron con asociación significativa para cada *Yi* de interés (criterios de asociación significativa tomados en cuenta: valor p < 0,25 o criterio de Hosmer-Lemeschow, plausibilidad teórica, y valor de *OR* u *RP* significativo, esto es, que el intervalo de confianza al 95% no incluyese el valor nulo o neutro de uno) fueron llevadas a modelos de regresión logística ordinal múltiple, calculando *RP* y *OR* ajustados por otras covariables, descartando fenómenos de confusión e interacción.

Se eligió como modelo final para el puntaje global HOME45 (*Y*) aquél que fuese parsimonioso, representativo, significativo, plausible de acuerdo con la literatura revisada y que demostrase un adecuado ajuste con una colinealidad limitada. Los análisis estadísticos se realizaron en el software RStudio Versión 1.2.5033^18^.

El proyecto de investigación fue aprobado por el Comité de Ética institucional de la Fundación Universitaria Autónoma de las Américas.

## Resultados

La población accesible identificada por censo e incluida en este estudio estuvo conformada por 76 niños y sus cuidadores. Con respecto a las variables sociodemográficas, el 55% (n=42) de los infantes pertenecían al sexo masculino, con una mediana de edad de 36 meses (RIQ=11, 12-48) para ambos sexos. De acuerdo con variables evaluadas en los cuidadores, la mediana de edad para estos fue de 35 años (RIQ=19, 19-85, p Shapiro-Wilk=0,00). Según nivel de escolaridad, 52,6% (n=40) terminaron el grado bachillerato, 18,42% (n=14) la primaria, el 11,8 % (n=9) tuvo educación superior de pregrado y 10,53% (n=8) cursó una carrera técnica.

Al indagar por variables ligadas a la dinámica familiar se encontró que el cuidado diario del niño fue brindado en el 67% (n=51) de los casos por la madre y un 17% (n=13) por la abuela. El 48,6% (n=37) de las familias eran nucleares, seguido de la familia extendida en un 44,7% (n=34) y monoparental 3,9% (n=3). Con respecto a la participación de la figura paterna en la educación y cuidado del infante, se reportó que un 47,3% (n=36) de los padres tenían interacciones mayores a 1 hora durante el día, y el 14,4% (n=11) refirieron no participar en dicho cuidado.

En el uso de estrategias de premio o corrección, el 61,8% (n=47) refirió utilizar el diálogo con el infante, el 19,7% (n=15) castigo físico y el 18,4% (n=14) castigo verbal. En relación con las formas de incentivar, el 92% (n=70) de los cuidadores refirió utilizar algún tipo de premio (regalos, salir al parque, jugar). Por otra parte, la toma de decisiones en la familia en un 63% (n=48) fueron responsabilidad de la madre, 17% del padre (n=13) y 7,8% (n=6) del abuelo.

Al evaluar variables relacionadas a los hábitos de lectura, se encontró que el 32,8% (n=25) de los cuidadores refirieron no leer a los niños durante la semana, el 28,9% (n=22) de una a dos veces en la semana. El 25% (n=20) de los niños tenían un libro o ninguno de su propiedad, y un 23,4% (n=19) de tres a cinco libros accesibles.

Al medir con el instrumento HOME45 el grado de estimulación total que recibía el niño en casa por parte de su cuidador principal, predominó la estimulación *“alta”* (*Y )* (50%, n=38), seguida por *“media”* (*Y )* (31,58%, n=24), y sólo un 18,42% (n=14) presentó *baja* estimulación (*Y*_*3*_ *)*. El análisis descriptivo de los puntajes totales de las dimensiones se detalla en la Tabla 2.

**Tabla 2:** Grado de estimulación en el hogar según dimensiones del inventario HOME45.

Dimensión	Frecuencia (n = 76)	%
Responsividad		
Alta	21	27,63%
Media	37	48,68%
Baja	18	23,68%
Organización		
Alta	54	71,05%
Media	13	17,10%
Baj	9	11,84%
Materiales de juego		
Alta	44	57,89%
Media	23	30,26%
Baja	9	11,84%
Variedad		
Alta	32	42,11%
Media	31	40,79%
Baja	13	17,11%
Aceptación		
Alta	22	28,95%
Media	26	34,21%
Baja	28	36,84%
Involucramiento		
Alta	32	42,11%
Media	33	43,42%
Baja	11	14,47%

En las Tablas 3, 4 y 5 se observa el análisis bivariado para cada categoría ordinal de *Y,* mostrando únicamente las asociaciones estadísticamente significativas y temáticamente relevantes. En la Tabla 5 se muestra el modelo final de regresión logística ordinal múltiple.

**Tabla 3:** Análisis bivariado para la variable dependiente como respuesta binaria según puntaje de estimulación global en el hogar, HOME45/Alta (*Y*_*1*_ *)*.

**Variables independientes (*Xi*)**	Categorías	**Home45 alta (*Y1*) (sí) n (%)** **n=38**	**Home45 alta (*Y1*) (no) n (%)** **n=38**	Valor p	OR (IC95%)
Estrategias de corrección	Castigo Físico	4 ( 10,5)	11 ( 28,9)	0,05*	N.A
	Castigo Verbal	10 ( 26,3)	4 ( 10,5)		
	Diálogo	24 ( 63,2)	23 ( 60,5)		
Lectura	Si	29 ( 76,3)	18 ( 47,4)	0.01**	3,58 (1,34 - 9,56)
Fiestas Infantiles	Si	28 ( 73,7)	18 ( 47,4)	0,02**	3,11 (1,19 - 8,15)
Paseos familiares	Si	31 ( 81,6)	24 ( 63, 2)	0,07**	2,58 (0,90 - 7,40)
Felicitar verbalmente	Si	30 ( 78,9)	23 ( 60,5)	0,08**	2,45 (0,89 - 6,75)
Responsividad ***	Alta	16 ( 42,1)	5 ( 13,2)	0,01**	4,80 (1,53 - 15,01)
	Baja	3 ( 7,9)	15 ( 39,5)	0,00*	0,13 (0,03 - 0,51)
Aceptación ***	Alta	19 ( 50,0)	3 ( 7,9)	0,00*	11,67 (3,06 - 44,54)
	Baja	9 ( 23,7)	19 ( 50,0 )	0,02**	0,31 (0,12 - 0,83)
Organización ***	Alta	31 ( 81,6)	23 ( 60,5)	0,04**	2,89 (1,01 - 8,23)
	Baja	1 ( 2,6 )	8 ( 21,1)	0,03 *	0,10 (0,01 - 0,86)
Materiales ***	Alta	34 ( 89,5)	10 ( 26,3)	0,00*	23,80 (6,73 - 84,14)
	Baja	0 (0,0)	9 ( 23,7 )	0,00*	0,04 (0,00 - 0,72)
Involucramiento ***	Alto	27 ( 71,1)	5 (1 3,2)	0,00*	16,20 (5,01 - 52,36)
	Bajo	2 ( 5,3 )	9 ( 23,7)	0,05*	0,18 (0,04 - 0,89)
Variedad ***	Alta	25 ( 65,8)	7 ( 18,4)	0,00**	8,52 (2,95 - 24,56)
	Baja	2 (5, 3)	11 ( 28,9)	0,01*	0,14 (0,03 - 0,67)

*Prueba exacta de Fisher, N.A=no aplica, **X2 de independencia ***Categoría de referencia: “media”.

**Tabla 4:** Análisis bivariado para la variable dependiente puntaje de estimulación global en el hogar según inventario HOME45/Media (*Y*_*2*_ *)*.

Variables	Categoría	**Home45 alta (*Y1*) (sí) n (%)** **n=24**	**Home45 alta (*Y1*) (no) n (%)** **n=52**	Valor p	OR (IC95%)
Cuidado diario por	Abuela/Abuelo	3 (12,5)	17 (32,7)	0,03*	NA
	Madre	21 87,5)	30 (57,7)		
	Padre	0 (0,0)	2 (3,8)		
	Tía	0 (0,0)	3 (5,8)		
Aceptación	Alta	2 (8,3)	20 (38,5)	0,01*	0,15 (0,03 - 0,69)
	Baja	13 (54,2)	15 (28,8)	0,03**	2,92 (1,07 - 7,94)
Materiales***	Alta	9 (37,5)	35 (67,3)	0,01**	0,29 (0,11 - 0,80)
	Media	15 (62,5)	8 (15,4)	0,00**	9,17 (3,00 - 28,04)
Involucramiento***	Alto	5 (20,8)	27 (51,9)	0.01**	0,24 (0,08 - 0,75)
	Medio	17 (70,8)	16 (30,8)	0,00**	5,46 (1,89 - 15,76)
Variedad****	Baja	7 (29,2)	6 (11,5)	0,05**	3,16 ( 1,03 - 10,74)

*Prueba exacta de Fisher, N.A=no aplica, **X2 de independencia ***Categoría de referencia: “baja” ****Categoría de referencia: “alta”

**Tabla 5:** Análisis bivariado para la variable dependiente puntaje de estimulación global en el hogar según inventario HOME45/Baja (*Y*_*3*_ ).

Variables	Categoría	**Home45 alta (*Y1*) (sí) n (%)** **n=14**	**Home45 alta (*Y1*) (no) n (%)** **n=62**	Valor p	OR (IC95%)
Lectura	Si	2 (14,3)	45 (72,6)	0,00*	0,06 (0,01 - 0,31)
Fiestas Infantiles	Si	3 (21,4)	43 (69,4)	0,00*	0,12 (0,03 - 0,48)
Paseos familiares	Si	7 (50,0)	48 (77,4)	0,04**	0,29 (0,09 - 0,97)
Responsividad	Alta	0 (0,0)	21 (33,9)	0,01*	0,07 (0,00 - 1,17)
	Baja	9 (64,3)	9 (14,5)	0,00**	10,60 (2,88 - 38,96)
Aceptación	Alta	1 (7,1)	21 (33,9)	0,06*	0,15 (0,02 - 1,23)
	Alta	6 (42,9)	48 (77,4)	0,01**	0,22 (0,06 - 0,74)
Organización	Baja	5 (35,7)	4 (6,5)	0,01*	8,06 (1,81 - 35,76)
	Alta	1 (7,1)	43 (69,4)	0,00*	0,03 (0,00 - 0,28)
Materiales	Baja	9 (64,3)	0 (0,0)	0,00*	215,91 (11,03 - 4227,37)
	Alto	0 (0,0)	32 (51,6)	0,00*	0,03 (0,00 - 0,57)
Involucramiento	Bajo	7 (50,0)	4 (6,5)	0,00*	14,50 (3,38 - 52,28)
	Alta	0 (0,0)	32 (51,6)	0,00*	0,03 (0,00 - 0,57)
Variedad**	Media	10 (71,4)	21 (33,9)	0,02*	4,88 (1,37 - 17,44)

*Prueba exacta de Fisher, N.A=no aplica, **X2 de independencia **Categoría de referencia:“Baja”.

Para *Y*_*1*_ , cuando los cuidadores no leen a los niños, se reduce hasta en un 71% la oportunidad de presentar **alta estimulación**, en comparación con aquellos niños cuyos cuidadores sí les incentivan el hábito de la lectura. Por otra parte, cuando los cuidadores no llevan a los niños a fiestas infantiles, se reduce hasta en un 76% la oportunidad de presentar este desenlace, en comparación con aquellos niños que son incentivados para participar en fiestas infantiles por parte de sus cuidadores. Adicionalmente, por cada año de edad adicional por encima de la mediana (35 años) que tenga el cuidador, se reduce hasta en un 5% la oportunidad de presentar el desenlace, en comparación con aquellos niños con los cuidadores más jóvenes. Además, cuando los cuidadores emplean el castigo verbal o físico, se reduce hasta en un 84% la oportunidad de presentar alta estimulación, en comparación con aquellos niños cuyos cuidadores emplean el diálogo.

Para *Y*_*2*_ , cuando el puntaje de la diada niño (a)/cuidador es “media” en la dimensión “materiales”, se incrementa hasta 21 veces la oportunidad de presentar **estimulación media**, en comparación con aquellas diadas con puntajes bajos o altos. Por otra parte, cuando el puntaje de la diada niño(a)/cuidador es “bajo” en la dimensión “variedad”, se reduce hasta en un 83% la oportunidad de presentar este desenlace, en comparación con aquellas diadas con puntajes altos o medios. Adicionalmente, cuando la toma de decisiones en el hogar es conjunta entre padre y madre, se reduce hasta en un 98% la oportunidad de presentar este desenlace, en comparación con aquellos niños en cuyos hogares las decisiones son tomadas por solamente uno de los padres o por otro familiar. Además, se encontró una interacción positiva significativa entre “Involucramiento media” y “Variedad media”, lo cual pone de manifiesto que, tener un puntaje medio en la dimensión “involucramiento” sólo incrementa la probabilidad de presentar **estimulación media** cuando también se acompaña de una “Variedad media”. Y cuando estas dos variables (involucramiento y variedad) se presentan en conjunto en una misma diada, se incrementa hasta en 29 veces la oportunidad del desenlace, en comparación con las diadas que no presentan de manera conjunta puntajes medios en las dimensiones “involucramiento” y “variedad”.

Para *Y*_*3*_ , cuando los cuidadores no leen a los niños, se incrementa hasta en 16 veces la odds de presentar **estimulación baja**, en comparación con aquellos niños cuyos cuidadores sí les leen o incentivan el hábito de la lectura. Por otra parte, cuando una diada obtiene un puntaje bajo en la dimensión “Responsividad”, se incrementa hasta en 9,5 veces la odds de presentar este desenlace, en comparación con aquellas diadas que obtuvieron puntajes medios o altos. Adicionalmente, cuando los cuidadores no incentivan la participación de los niños(as) en fiestas infantiles, se incrementa hasta en 9 veces la odds de presentar el desenlace, en comparación con aquellos niños cuyos cuidadores sí los llevan a fiestas infantiles. Además, cuando una diada obtiene un puntaje bajo en la dimensión “Organización”, se incrementa hasta en 16 veces la odds de presentar el desenlace, en comparación con aquellas diadas que obtuvieron puntajes altos o medios.

Todos los efectos de cada uno de los predictores independientes (*Xi*) sobre cada desenlace (*Yi*) fueron estadísticamente significativos, ajustando por las demás covariables incluidas en las ecuaciones finales, permaneciendo estas constantes y produciéndose una vez se ha ajustado por fenómenos de confusión e interacción. Adicionalmente, los indicadores de ajuste del modelo final elegido, clasificación correcta, validez global y capacidad de explicación de la varianza total fueron aceptables, y el modelo elegido pudo considerarse como parsimonioso (ver Tabla 6).

**Tabla 6:** Regresión logística ordinal múltiple, para: HOME45 alta (*Y*_*1*_ ) (prevalencia global= 50%), HOME45 media (*Y*_*2*_ ) (prevalencia global= 31,58%), HOME45 baja (*Y*_*3*_ ) (prevalencia global= 18,42%) N=76.

**Variable independiente (*X1*)**	**Alta vs media y baja (*Y1*)**		**Media vs alta y baja (*Y2*)**		Baja vs media y alta (Y_3_)	
	RP (IC95%)	Valor p	RP (IC95%)	Valor p	RP (IC95%)	Valor p
Lectura (no)	0,29 (0,09 - 0,87)	0,01	-	-	14,85 (3,21 - 108,07)	0,00
Fiestas infantiles (no)	0,24 (0,07 - 0,79)	0,04	-	-	7,44 (1,44 - 57,49)	0,03
Edad del cuidador (mayor a 36 años)	0,95 (0,92 - 0,99)	0,01	-	-	-	-
Estrategias de corrección (castigo verbal o físico)	0,16 (0,03 -0,98)	0,12	-	-	-	-
Materiales (media)	-	-	21,16 (4,12 -108,74)	0,00	-	-
Variedad (baja)	-	-	0,17 (0,02 - 1,16)	0,60	-	-
Toma de decisiones (Padre y Madre)	-	-	0,02 (0,00 - 1,04)	0,19	-	-
Involucramiento Media*Variedad	-	-	28,9 (1,42 - 586,2)	0,02	-	-
media						
Responsividad (baja)	-	-	-	-	8,35 (1,62 - 59,40)	0,00
Organización (baja)	-	-	-	-	13,31 (1,77 - 163,45)	0,02

Valor p prueba ómnibus = 0,00, Valor p bondad de ajuste de Hosmer - Lemeshow = 1,00

Todas las variables contribuyeron significativamente al ajuste del modelo según la prueba ANOVA con estadístico *X*2, valor p < 0,05. VIF = factor de inflación de la varianza para evaluar colinealidad. Para todas las variables la colinealidad fue menor de 5.

Ecuaciones de regresión logística ordinal:

*Probabilidad de Y*_*1*_ = 1/1+e-(4,34-1,25(Lectura(no))-1,42(Fiestas infantiles(no))-0,05(Edad del cuidador mayor a 36 años)-1,82(Estrategias de corrección (castigo verbal o físico))) *Probabilidad de Y*_*2*_ = 1/1+e-(-0,22+3,05(Materiales (media))-1,79(Variedad (baja))-3,80 (Toma de decisiones (Padre y Madre))+3,36 (Involucramiento media*Variedad media))

*Probabilidad de Y*_*3*_ = 1/1+e-(-4,24+2,75 (Lecturas (no))+2,25(Responsividad(Baja))+2,17(Fiestas Infantiles (no))+2,76(Organización(Baja)))

Indicadores de ajuste y significancia del modelo final:

AIC = 95,65 para Y1, 73,86 para Y2, 46,61 para Y3.

R2 McFadden = 33,13% para Y1, 56,25% para Y2, 61,31% para Y3.

Porcentaje de Clasificación correcta del modelo = 54,14% para Y_1_, 91,93% para Y_2_, 100,20% para Y3

IVG (Índice de Validez Global) del modelo= 81,24% para Y_1_, 90,96% para Y_2_, 91,84% para Y_3_.

Deviance del modelo= -2llh modelo nulo = 105,36 (gl=75) para Y_1_, 94,79 (gl=75) para Y_2_, 72,61 (gl=75) para Y_3_

-2llh modelo ajustado = 83,65 (gl=70) para Y1, 55,85 (gl=67) para Y2, 36,61 (gl=71) para Y3.

## Discusión

Los resultados de este estudio representan un aporte a las necesidades de abordaje en áreas del desarrollo infantil en sus primeras etapas, con una exploración en este caso a los niños, niñas y cuidadores que se encuentran vinculados a un programa de salud pública de primera infancia que integra manejo educativo institucional y familiar.

Al indagar sobre factores sociodemográficos de la población, la edad del cuidador en este estudio tuvo una media de 35 años, similar a lo reportado por Bedregal con una media de edad de 33,9. En lo referente a nivel de escolaridad un 56% de los cuidadores culminaron el bachillerato alcanzando en promedio 11 años en curso, comparable con lo reportado por este mismo autor en donde los años de estudio de la población estuvieron en 9,8 años cursados^19^.

La variable escolaridad es determinante cuando se habla de estimulación del desarrollo, de hecho el nivel de escolaridad de las madres es un aspecto que influye de forma notoria en el desarrollo cognitivo infantil^20^, de tal forma un estudio realizado en las comunas de Medellín, Colombia reportó que entre los factores que explican las dificultades cognitivas de los niños, los antecedentes de problemas académicos de la madre son especialmente relevantes^21^ y se establecen como un poderoso predictor del coeficiente intelectual del niño^22^.

El 48,6% de las familias participantes en el estudio eran nucleares, hallazgo evidenciado por Luque^23^, quien demostró cómo la condición nuclear de la familia contribuye en el desarrollo intelectual del niño dado que el factor afectivo en la dinámica de este tipo de familia resulta mucho más favorable a diferencia de otro tipo de familias.

Se evidenció además que el cuidado del niño en general fue brindado por la madre en el 67% de los casos, y 17% la abuela, similar a lo reportado por Bedregal, quien encontró que la atención de los niños generalmente estaba a cargo de un cuidador mujer (76,4%). Así mismo, un estudio realizado en Bucaramanga, Colombia evidenció hallazgos similares (cuidador mujer 95%)^19^^,^^24^. Adquiere especial relevancia que el cuidado esté a cargo de la madre, debido a que un comportamiento sensible, responsivo y con coherencia disciplinar frente a los requerimientos de los hijos, promueve mayores niveles de desarrollo físico, intelectual y emocional en estos, a la vez que enriquece la relación de apego seguro^25^^,^^26^.

Con respecto a la participación del padre en los procesos de desarrollo del niño, este estudio reveló que un 47,3% de los padres tienen interacciones mayores a 1 hora durante el día, y el 14,4% refirieron no participar en dicho cuidado. Esto es comparable con otra investigación que buscó medir la prevalencia de la estimulación paterna y el involucramiento en la crianza, en la cual el total de los padres estaban muy involucrados en las actividades de estimulación (5 o 6 actividades). Las nuevas dinámicas familiares evidencian de forma clara la necesidad del apoyo de otros miembros de la familia en la crianza y cuidado^27^^,^^43^.

La tipología familiar afecta de manera potencial los procesos de socialización. La transmisión de las prácticas de crianza relacionadas con el castigo, explica la repetición de modelos tomados de los abuelos o abuelas y/o o las madres y los padres^28^.

En el aspecto relacionado con el nivel de estimulación que los niños reciben, este estudio mostró que el 50% de los hogares eran estimulantes para el desarrollo. Una investigación realizada en Argentina mostró que en un grupo de niños que se desarrollaron con parámetros adecuados, el 34% provenían de hogares no estimulantes (n = 56), mientras que el 66% lo hacían de hogares estimulantes. Este mismo mostró que el 6% del total de los niños evaluados presentaron sospecha de padecer retraso global del desarrollo en ambientes poco estimulantes y con baja calidad educativa materna. Por otro lado, el 20% (n=43), se desarrollaron típicamente aún en las mismas condiciones de estímulo y calidad educativa^29^. Contrario a esto, otra investigación realizada en Perú que buscó asociar estimulación en el hogar y desarrollo psicomotor en niños de 3 años, reportó que el 72% tenían ambiente inadecuado de estimulación, asociado a retrasos en el desarrollo psicomotor en 37,5% de los casos^30^.

Contrastando con nuestro estudio, una investigación realizada en México que evaluó el nivel de estimulación de un grupo de niños pertenecientes a un programa de vigilancia de desarrollo, evidenció que en los no expuestos al programa predominó la baja estimulación (49%), mientras que en los expuestos la alta estimulación tuvo un mayor predominio (35,9%)^31^.

En lo referente a la lectura, este estudio evidenció que el 32,8% de los cuidadores refirieron no leer a los niños y un 25% no contaban con libros propios de su edad. Un informe de la UNICEF en el año 2016 reportó que menos de la mitad de los niños entre 3 a 5 años de la mayoría de los países disponen de tres libros o más apropiados para su desarrollo lector^32^.

Un estudio realizado en Singapur que exploró la relación entre el entorno de alfabetización en el hogar y hábitos lectores del niño encontró que este acompañamiento puede apoyar el desarrollo de la alfabetización antes de la inserción del niño a la escuela y predice positivamente la competencia lectora^33^.

Con respecto a los resultados por dimensiones en el área de *aceptación* se evidenció bajo nivel de estimulación (36,84%), destacándose la necesidad de promoción de hábitos de lectura, dada su importancia para la formación de un vocabulario adecuado y procesamiento verbal, estudios recomiendan potenciar desde estadios iniciales de educación primaria escenarios donde los niños inicien o mantengan sus prácticas lectoras^34^^,^^35^^,^^36^.

Según este estudio la utilización del castigo físico o verbal como estrategia de corrección reduce hasta en un 84% las oportunidades de estimulación adecuada en los infantes. La bibliografía reporta que estas estrategias continúan utilizándose para manejar comportamientos inadecuados de los niños, considerándose sobre otras formas correctivas^37^^,^^38^. Este hallazgo sugiere la necesidad de trabajo con familias en temas que fomenten la disciplina saludable y métodos de corrección efectivos en la crianza positiva.

En lo referente a la edad del cuidador principal el modelo evidenció que a medida que aumenta la edad del cuidador se reduce la oportunidad de ofrecer ambientes estimulantes en comparación con los niños que son atendidos por cuidadores jóvenes. Sin embargo, no se encontró literatura que apoyara este hallazgo, pero a los autores nos parece relevante este resultado dado que los nuevos estilos de vida e inserción de la mujer al mercado laboral conlleva a una maternidad tardía en algunos casos. Con respecto a la edad de los cuidadores el estudio de Jorge reportó que los padres mayores de 40 años acostumbran el uso de pautas educativas cambiantes, ausencia del diálogo e interacción asertiva con el infante^39^.

En la subescala que mide *responsividad* por parte del cuidador, se evidenció como significativo que los puntajes bajos para esta categoría incrementan las posibilidades de una estimulación inadecuada, esto marca la relevancia de la conexión emocional y la atención que el cuidador debe presentar ante las conductas del niño. Así mismo, estudios han mostrado que la *responsividad* impacta en el desarrollo verbal y no verbal. De esta manera, la respuesta responsiva y sensible por parte del cuidador primario tiene gran peso sobre los procesos cognitivos así como en procesos de autoregulación en la conducta del infante^40^^,^^41^.

Por otra parte al evaluar el material didáctico y lúdico, se destaca que los hábitos lectores de los niños pueden ser predictores del desarrollo, de esta manera, de acuerdo con nuestro modelo, cuando los cuidadores no leen a los niños se reduce hasta en un 71% la oportunidad de presentar una estimulación alta. El estudio de Gago que buscó la asociación entre un ambiente estimulante y capacidades de regulación de los niños mostró que a mayor frecuencia de las actividades de lectura en el hogar se presenta mayor capacidad de regulación en la conducta de los niños y mayores tiempos de concentración en las tareas^42^.

Como limitaciones del estudio se reconocen: el tamaño de muestra escaso, la presencia de pocas unidades en algunas categorías de comparación y cierta colinealidad (VIF entre 1 y 5) para algunas variables, lo cual podría sobreestimar la asociación.

## Conclusiones

El nivel de estimulación recibido por los infantes del estudio fue Alto-medio, se destacaron conductas que favorecían la estimulación alta, como felicitar al niño(a), corregirlo mediante diálogo, incentivar: lectura, paseos familiares y participación en fiestas infantiles, además de la tipología familiar y la edad del cuidador, son variables relacionadas con la estimulación global y con sus dimensiones. Se sugiere educación en áreas que incentiven la lectura, el cual se marcó como un hallazgo a trabajar entre las familias.
